# Reciprocal Regulation of Mitofusin 2-Mediated Mitophagy and Mitochondrial Fusion by Different PINK1 Phosphorylation Events

**DOI:** 10.3389/fcell.2022.868465

**Published:** 2022-05-12

**Authors:** Jiajia Li, Xiawei Dang, Antonietta Franco, Gerald W Dorn

**Affiliations:** Center for Pharmacogenomics, Department of Internal Medicine, Washington University School of Medicine, St. Louis, MO, United States

**Keywords:** MFN2, mitochondrial quality control, fusion, mitofusin regulation, Parkin, PINK1 kinase, phosphorylation

## Abstract

Mitochondrial repair is essential to metabolic homeostasis. Outer mitochondrial membrane mitofusin (MFN) proteins orchestrate mitochondrial fusion that opposes mitochondrial degeneration caused by senescence. Depending upon physiological context, MFN2 can either mediate mitochondrial fusion or recruit cytosolic Parkin to initiate mitophagic elimination. Because it is not clear how these events are counter-regulated we engineered and expressed MFN2 mutants that mimic phosphorylated or non-phosphorylatable MFN2 at its PINK1 phosphorylation sites: T111, S378, and S442. By interrogating mitochondrial fusion, polarization status, and Parkin binding/mitophagy as a function of inferred MFN2 phosphorylation, we discovered that individual MFN2 phosphorylation events act as a biological “bar-code”, directing mitochondrial fate based on phosphorylation site state. Experiments in *Pink1* deficient cells supported a central role for PINK1 kinase as the pivotal regulator of MFN2 functionality. Contrary to popular wisdom that Parkin-mediated ubiquitination regulates MFN-mediated mitochondrial fusion, results in *Prkn* null cells demonstrated the dispensability of Parkin for MFN2 inactivation. These data demonstrate that PINK1-mediated phosphorylation is necessary and sufficient, and that Parkin is expendable, to switch MFN2 from fusion protein to mitophagy effector.

## Introduction

Mitochondria and their host cells co-exist in a mutually dependent relationship ∼1.5 billion years old (Sagan 1967; Schwartz and Dayhoff, 1978; Yang et al., 1985). The consequence of primitive single cell organism invasion by ancient archaea (Zaremba-Niedzwiedzka et al., 2017) was an enduring endosymbiotic affiliation whose evolutionary legacy is the familiar modern organelle-cell interaction. As with any marriage of independent entities, convergence of mitochondria and host cells involves give and take. Thus, mitochondria gave ∼99% of their original genomes to their hosts, and the cells took responsibility for transcribing and translating nuclear-encoded mitochondrial proteins. Mitochondrial biogenesis, which is the culmination of replicative mitochondrial fission, synthesis of the 13 mtDNA-encoded respiratory chain proteins and incorporation of ∼1,000 nuclear-encoded mitochondrial proteins (Jornayvaz and Shulman, 2010), therefore requires exquisite coordination between individual members of the mitochondrial collective and the host cell. The same is true of mitophagy, the selective elimination of self-identified senescent or damaged mitochondria by the cellular autophagy system (Ashrafi and Schwarz, 2013).

The above paradigm supports a central role for mitochondrial fission in mitochondrial quantity and quality control, i.e., in biogenesis and mitophagy, respectively (Kowald and Kirkwood, 2011; Twig and Shirihai, 2011; Chen and Chan, 2017). Healthy mitochondria replicate through symmetric fission wherein the parent organelle gives rise to two healthy daughters. By contrast, an impaired parent organelle can undergo asymmetric fission to rid itself of damaged components that are sequestered into one of the daughter organelles. The smaller depolarized daughter containing damaged parental components is targeted for mitophagic removal whereas the remaining healthy daughter fuses with and rejoins the cellular mitochondrial collective. Controlled counter-regulation of mitochondrial fusion and fission is essential for these processes to occur in a manner that is orderly and maintains mitochondrial homeostasis. Here, we asked how mitochondrial fusion is regulated and sought to define the consequences of regulated mitochondrial fusion on mitophagy.

The initial events in mitochondrial fusion, i.e., reversible tethering of organelles followed by irreversible fusion of outer mitochondrial membranes, are mediated by mitofusin (MFN) proteins (Chan, 2006; Song et al., 2009). Conventional wisdom holds that mitochondrial fusion is negatively regulated, and mitophagy is positively affected, by selective proteasomal degradation of mitofusins following their ubiquitination by Parkin (Neutzner and Youle, 2005; Cohen et al., 2008; Gegg et al., 2010; Poole et al., 2010; Glauser et al., 2011; Karbowski and Youle, 2011; McLelland et al., 2018). Importantly, Parkin recruitment to mitochondria is facilitated by MFN2 after its phosphorylation by PINK1 kinase (Shiba-Fukushima et al., 2012; Chen and Dorn, 2013; Gong et al., 2015; Rocha et al., 2018), which also phosphorylates both Parkin and its ubiquitin substrate (Kane et al., 2014; Koyano et al., 2014). This raised the possibility that PINK1-mediated MFN phosphorylation itself regulates MFN functionality, in anticipation of subsequent ubiquitination. Our goal here was to disentangle these complex relationships and better understand how PINK1-mitofusin interactions orchestrate mitochondrial fate.

## Materials and Methods

### Cell Lines

ATCC (Manasses Virginia, United States of America) was the source for large T antigen immortalized murine embryonic fibroblasts (MEFs) derived from *Mfn1* knockout (CRL-2992), *Mfn2* knockout (CRL-2994), and *Mfn1/Mfn2* double knockout mice (CRL-2993) and HEK 293 cells (CRL-1573). Cells were maintained in Dulbecco’s modified eagle’s medium (Thermo Fisher Scientific, 11965-084) supplemented with 10% fetal bovine serum (Thermo Fisher Scientific, 26140-079), 1x non-essential amino acids (Thermo Fisher Scientific, 11130-051), 100 U/ml penicillin and 100 mg/ml streptomycin (Thermo Fisher Scientific, 15140-122) at 37°C with 5% CO_2_. The character of all cell lines was validated by immunoblot analysis.

FCCP (Carbonyl cyanide 4-(trifluoromethoxy)phenylhydrazone, Sigma, C2920) was used to stimulate mitophagy at a working concentration of 5 µM for 1 hour (for Parkin aggregation studies) or 8 hours (for mitoQC studies).

### Mouse Models

Primary MEFs were prepared as described (Song et al., 2017) from E13.5 embryos of B6.129S4-Pink1^tm1Shn^/J mice (Kitada et al., 2007) (The Jackson Laboratory #017946; *Pink1* KO MEFs) or B6.129S4-Prkn^tm1Shn^/J mice (Goldberg et al., 2003) (The Jackson Laboratory #006582; *Prkn* KO MEFs) and validated by PCR genotyping. Mouse studies were approved by the Washington University in St. Louis School of Medicine Animal Studies Committee, IACUC protocol number 19–0910 (Exp:12/16/2022).

### Protein Modeling

Hypothetical structures of human MFN2 were generated using Chimera UCSF. The putative closed conformation is based on structural homology with bacterial dynamin-like protein (PDB: 2J69) (Low and Löwe, 2006).

### DNA Plasmids and Adenoviral Expression Constructs

Su9-EGFP (Addgene, 23214) and mCherry-Parkin (Addgene, 23956) plasmids were purchased from Addgene.

Adenovirus β-galactosidase was purchased from Vector Biolabs (Vector Biolabs, #1080). Adenoviri expressing FLAG-MFN2 and its phosphorylation site mutants were constructed by Vector Biolabs. Adenovirus mCherry-Parkin was a generous gift from Dr. Åsa Gustafsson at University California San Diego.

MFN2 phosphorylation site mutants were generated from human MFN2 cDNA with an amino terminal FLAG epitope (Chen and Dorn, 2013) using the QuikChange Site-Directed Mutagenesis Kit (Agilent, 200519).

Mutagenesis primers used are listed below; mutation sites are indicated in bold:

Mfn2-T111A fw: gag​caa​tgg​gaa​gag​c**g**ccg​tga​tca​atg​cca​t
Mfn2-T111A rv: atg​gca​ttg​atc​acg​g**c**gct​ctt​ccc​att​gct​c
Mfn2-T111E fw: gac​gag​caa​tgg​gaa​gag​c**gag**gtg​atc​aat​gcc​atg​ctc​t
Mfn2-T111E rw: aga​gca​tgg​cat​tga​tca​c**ctc**gct​ctt​ccc​att​gct​cgt​c
Mfn2-S378A-fw: gac​tca​tca​tgg​ac**g**ccc​tgc​aca​tgg​cg
Mfn2-S378A-rv: cgc​cat​gtg​cag​gg**c**gtc​cat​gat​gag​tc
Mfn2-S378D-fw: cga​ctc​atc​atg​gac**ga**cct​gca​cat​ggc​ggc
Mfn2-S378D-rv: gcc​gcc​atg​tgc​agg**tc**gtc​cat​gat​gag​tcg
Mfn2-S442A fw: aga​tca​ggc​gcc​tc**g**ctg​tac​tgg​tgg​ac
Mfn2-S442A rv: gtc​cac​cag​tac​ag**c**gag​gcg​cct​gat​ct
Mfn2-S442E fw: gag​gag​atc​agg​cgc​ctc**gaa**gta​ctg​gtg​gac​gat​tac
Mfn2-S442E rv: gta​atc​gtc​cac​cag​tac**ttc**gag​gcg​cct​gat​ctc​ctc

All plasmids were transfected using Lipofectamine^TM^ 3000 Transfection Reagent (Thermo Fisher Scientific, L3000001) for 2 days following manufacturer's protocol. All adenoviruses were added at a MOI of 50 for 2 days.

### Image Analysis

All live-cell images were acquired using Nikon Ti confocal microscope equipped with a 60x 1.3NA oil immersion objective, in Krebs-Henseleit buffer.

Mitochondrial aspect ratio is the ratio of mitochondrial length/width. In HEK293 cells, mitochondria were visualized with Su9-EGFP plasmid encoding mitochondria-localized green fluorescent protein. In MEFs, mitochondria were visualized with mitoTracker Green (Thermo Fisher Scientific, M7514), a green fluorescent mitochondrial dye that stains mitochondria regardless of membrane potential. The aspect ratio of each cell was calculated as an average of at least twenty mitochondria length/width ratio using ImageJ.

To assess mitochondrial polarization status, mitochondria were visualized with MitoTracker Green and polarized mitochondria were visualized with TMRE (Tetramethylrhodamine, ethyl ester, Thermo Fisher Scientific, T669), a red dye that specifically stains mitochondria in a membrane potential-dependent manner. Mitochondrial depolarization was calculated as % of mitochondria without TMRE staining compared to that of all MitoTracker Green stained mitochondria.

For detection of Parkin localization to mitochondria, HEK293 cells were transfected with mCherry-Parkin, a red fluorescent protein-tagged Parkin. In MEFs, mitophagy was measured separately either as Parkin aggregation or mitolysosome formation using adenoviral vectors expressing either mcParkin or MitoQC, respectively. MEFs were transduced with adeno-mCherry-Parkin to visualize Parkin aggregation, calculated as % of cells with mCherry-Parkin clumps compared to that of all cells. In this system Parkin aggregates form at mitochondria (*vide infra*).

For quantification of mitoQC, MEFs were transduced with adeno-mitoQC encoding a reporter protein with mitochondria-localized tandem mCherry and EGFP proteins. Both proteins fluoresce at neutral pH, but after mitochondria are delivered to acidic lysosomes the green signal is quenched. MitoQC positivity is calculated as red-only organelles, reported as number of mitolysosomes per cell using the ImageJ plugin “mitoQC counter” (Montava-Garriga et al., 2020).

### Immunoblot Analysis

Whole cell lysates were harvested using Cell lysis buffer (Thermo Fisher Scientific, FNN0011) with 1 mM PMSF, protease inhibitor (Roche, 05892970001) and phosphatase inhibitor (Roche, 04906837001) following manufacturer’s protocol. Proteins were quantified with protein assay dye reagent concentrate (Bio-Rad, 500-0006) using SpectraMax M5^e^ spectrophotometer (Molecular Devices). Equal amounts of protein (30ug) were denatured by boiling at 95° for 10 min in 2x Laemmli sample buffer (Bio-Rad, 1610737) supplemented with β-mercaptoethanol (Sigma, S3148). Protein samples were size-separated on 4–15% pre-cast SDS-PAGE gels (Bio-Rad, 4561084, 5671084), transferred to PVDF membranes (GE Healthcare, 10600021) at 4°C using 110 mV for an hour in 1x transfer buffer containing 20% methanol (Thermo Fisher Scientific, A412P-4). Membranes were blocked with 5% nonfat dry milk (Bio-Rad, 1706404) in 1x phosphate-buffered saline, 0.05% Tween-20 (PBS, Sigma, P5368; Tween-20, Promega, and H5152) at room temperature for 30 min, incubated with primary antibodies diluted in blocking buffer (room temperature for 2 h or 4°C for overnight), followed by incubation with horseradish peroxidase (HRP)-conjugated secondary antibodies in blocking buffer at room temperature for 1 h. Immunoblot signals were visualized using the ECL western blotting detection reagent (Bio-Rad, 1705060) and a LI-COR Odyssey imaging system. Some immunoblot membranes were re-probed after stripping with One Minute western blot stripping buffer (GM Biosciences, GM6001).

Primary antibody against Mfn2 (Abcam, ab56889, 1:1,000) is from Abcam. Primary antibody against Mfn1 (Santa Cruz, sc50330, 1:1,000, or Abcam, ab126575, 1:200) is from Santa Cruz/Abcam. Primary antibody against β-actin (Proteintech, 66009-1-lg, 1:2000) is from Proteintech. HRP-linked secondary antibodies anti-mouse IgG (Cell Signaling Technology, cs7076, 1:2000) and anti-rabbit IgG (Cell Signaling Technology, cs7074, 1:2000) are from Cell Signaling Technology.

### Statistical Analysis

Data are presented as means ± SEM. Sample number (n) is the number of independent biological replicates, defined as studies performed at different times using different aliquots of cells. Each value represents the average of between 6 and 30 cells (for aspect ratio, depolarization and MitoQC) or between 80 and 200 cells (for Parkin aggregation), analyzing between 15 and 30 mitochondria per cell. Statistical comparisons used one-way ANOVA with Tukey’s post hoc test for multiple comparison among groups or *t*-test for paired comparisons. A *p* value of <0.05 was defined as significant.

## Results

### MFN2 T111, S378, and S442 Phosphorylation Status Modulates Mitochondrial Fusion, Respiratory Fitness and Parkin Recruitment

**FIGURE 1:**
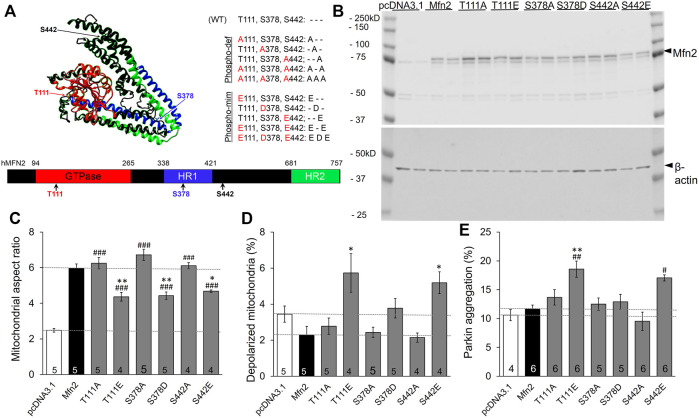
Effects of individual PINK1 phosphorylation site status on MFN2-modulated mitochondrial endpoints. **(A)** 3D (top) and 2D (bottom) depictions of human MFN2 and the positions of validated PINK1 phosphorylation sites. Domains are color coded. To the right are contemplated phospho-deficient (-def) and phospho-mimetic (-mim) engineered mutants. **(B)** Immunoblot analysis of MFN2 PINK1 phosphorylation site mutant expression in transfected HEK293 cells. Results from two independent transfections are shown. The MFN2 amino terminal FLAG epitope, which does not alter MFN2 functioning (Gong et al., 2015), slightly retards SDS-PAGE mobility of the expressed proteins compared to endogenous HEK293 MFN2. **(C–E)**. Change in mitochondrial aspect ratio **(C)**, depolarization **(D)** and Parkin recruitment **(E)** in HEK293 cells transfected with PINK1 phosphorylation site mutants. Empty pcDNA3.1 plasmid is negative control; wild-type MFN2 is positive control (dashed lines). N shown is number of independent experiments, each averaging results from 6-30 cells. Mutant vs. WT Mfn2: *p* < 0.05 *; *p* < 0.01 **; *p* < 0.001***; Mutant vs. pcDNA3.1: *p* < 0.05 #; *p* < 0.01 ##; *p* < 0.001 ###, all by ANOVA. Error bars are SEM.

Published mass spectroscopy results of *in vitro* MFN2 phosphorylation by PINK1 kinase identified T111, S378, and S442 as phosphorylation sites (Figure 1A) (Rocha et al., 2018). We performed site-directed mutagenesis at each of these sites to substitute either non-phosphorylatable alanine (Ala, A) or negatively charged glutamic acid (Glu, E) at sites T111 and S442 or aspartic acid (Asp, D) at S378, which mimic phosphorylation. These single phosphorylation site MFN2 mutants were transiently expressed in HEK293 cells (Figure 1B) and their effects assessed on the following endpoints: mitochondrial aspect ratio (organelle length/width) that increases with mitochondrial fusion (Vives-Bauza et al., 2010), mitochondrial polarization that dissipates with uncoupling of respiratory enzymes from oxidative phosphorylation (Crowley et al., 2016), and Parkin aggregation at mitochondria, reflecting the initiating step of mitophagy mediated via the PINK1-Parkin pathway (Durcan and Fon, 2015).

The baseline mitochondrial aspect ratio of HEK 293 cells (i.e., cells transfected with empty pcDNA3.1 plasmid) was 2–3 (Figure 1C white bar). Overexpressing wild-type (WT) MFN2 promoted mitochondrial elongation by enhancing mitochondrial fusion, increasing mitochondrial aspect ratio to ∼6 (Figure 1C black bar, MFN2). Substituting any of the three PINK1 phosphorylation sites with non-phosphorylatable alanine (Ala, A) was no different than WT MFN2 (Figure 1C). Conversely, mimicking phosphorylation with a negatively charged acidic amino acid at any of the three PINK1 sites reduced aspect ratio compared to WT MFN2 (Figure 1C). These results demonstrate sufficiency of phosphorylation at MFN2 T111, S378, and S442 to suppress mitochondrial fusion.

Mitochondrial fusion promotes organelle repair that maintains integrity of the respiratory chain (Chan, 2006; Dorn, 2019). Impaired fusion has been associated with dissipation of the electrochemical gradient that drives oxidative phosphorylation and ATP production, referred to as mitochondrial depolarization (Detmer and Chan, 2007). Consistent with this paradigm, mimicking phosphorylation at T111 and S442, which had impeded fusion, provoked mitochondrial depolarization. However, pseudo-phosphorylation at S378, which had suppressed fusion to the same extent as mimicking phosphorylation at the other two sites, failed to provoke depolarization compared to WT MFN2. As with fusion, preventing phosphorylation by alanine substitution (A) had no effect compared to WT MFN2 (Figure 1D). Taken together, the results in Figures 1C,D dissociate the responses of mitochondrial depolarization and fusion as directed by MFN2 phosphorylation.

Normal polarization of mitochondria expressing MFN2 S378D is difficult to explain if failure of fusion-mediated mitochondrial repair is the mechanism for mitochondrial depolarization. We considered that mitochondrial depolarization is linked with mitophagy: loss of polarization stabilizes PINK1 kinase, enabling Parkin recruitment and its ubiquitination of outer mitochondrial membrane proteins, which attracts autophagosomes (Durcan and Fon, 2015). MFN2 can play a role in this process by recruiting Parkin after PINK1 kinase-mediated phosphorylation on T111 and S442 (Chen and Dorn, 2013). Our findings validated this notion, as Parkin aggregation at mitochondria (Supplementary Figure S1) was increased in cells expressing pseudo-phosphorylated MFN2 at these sites (E111 and E442; Figure 1E). Strikingly, neither mimicking MFN2 phosphorylation with S378D nor preventing phosphorylation at that site with S378A had any impact on mitochondrial Parkin recruitment (Figure 1E). Thus, mitochondrial depolarization and Parkin recruitment are concordantly modulated by MFN2 phosphorylation at PINK1 sites.

### Phosphorylation of MFN2 T111 and S442, but Not MFN2 S378, Regulates Mitochondrial Parkin Recruitment

Considering it unlikely that PINK1 phosphorylation of MFN2 can target single amino acids, we next defined the functional consequences of combinatorial phosphorylation events. To avoid confounding effects of endogenous mitofusins these studies were performed in murine embryonic fibroblasts (MEFs) lacking all endogenous mitofusins, i.e., *Mfn1/Mfn2* double knockout (*Mfn* DKO) MEFs.

**FIGURE 2:**
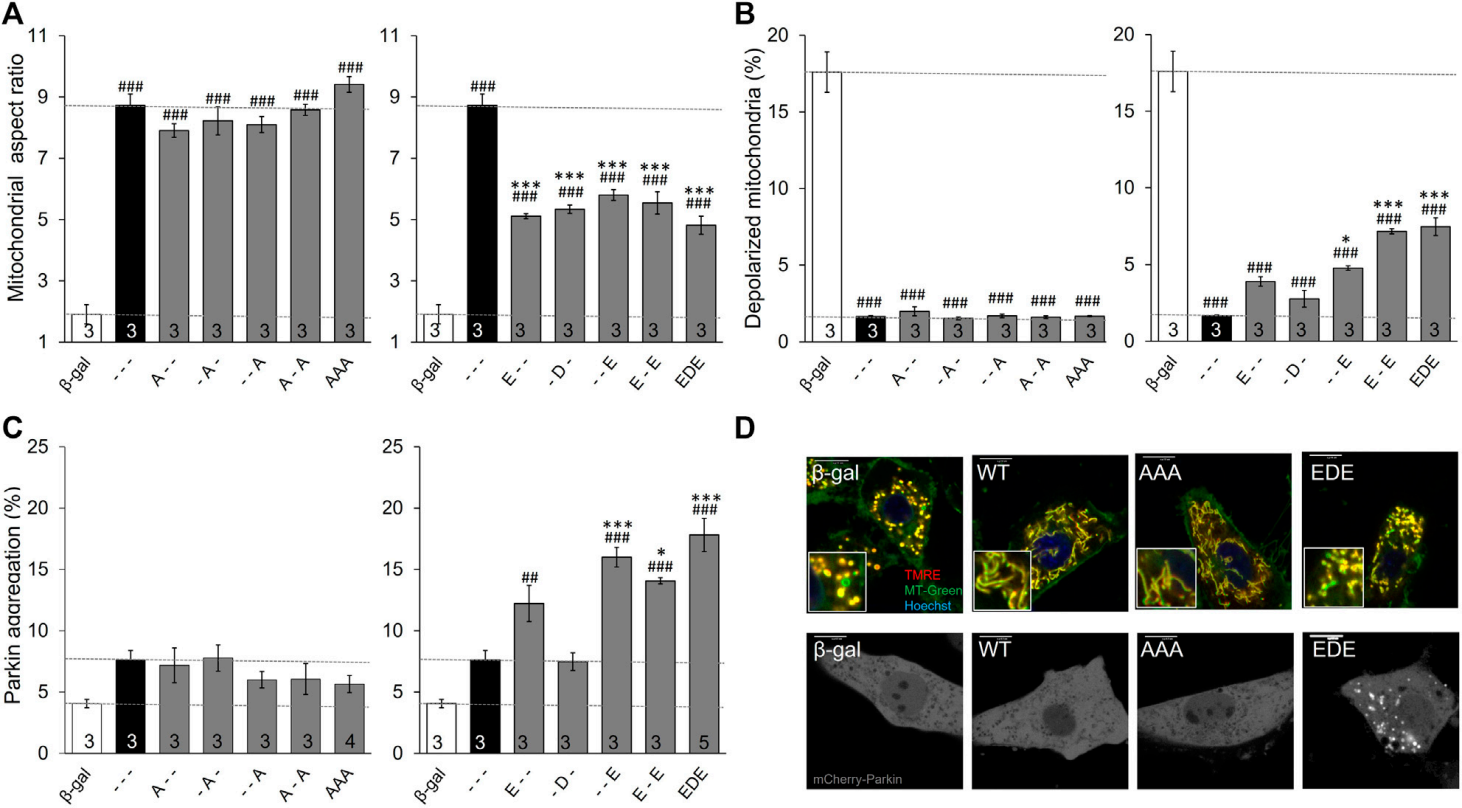
Intrinsic functioning of individual and combinatorial MFN2 PINK1 phosphorylation site mutants expressed in *Mfn1/Mfn2* double knockout MEFs. MFN2 mutants designated as in Figure 1A were expressed by adenoviral transduction of *Mfn1/Mfn2* double null murine fibroblasts. Adeno β-gal was negative control; --- indicates no mutation at T111, S378 or S442 (i.e., WT MFN2). **(A)** Mitochondrial aspect ratio. **(B)** Mitochondrial depolarization. **(C)** Parkin recruitment. Results for phosphorylation-defective (A substitutions) and phosphorylation mimic (E or D substitutions) are separated for clarity; β-gal and --- are duplicated for comparison. **(D)** Representative confocal images. N shown is number of independent experiments. Mutant vs. adeno WT Mfn2: *p* < 0.05 *; *p* < 0.01 **; *p* < 0.001***; MFN2 vs. adeno β-gal: *p* < 0.05 #; *p* < 0.01 ##; *p* < 0.001 ###, all by ANOVA. Error bars are SEM.

Three important observations accrued from studying MFN2 phosphorylation site mutants in *Mfn* DKO cells. First, preventing phosphorylation of MFN2 at any of the three sites, or even all three simultaneously (MFN2 AAA), did not alter mitochondrial aspect ratio, depolarization, or Parkin recruitment relative to WT MFN2 (Figures 2A–C, left panels). This indicates that the basal state of MFN2 in healthy cells is unphosphorylated at these sites. Second, as in HEK293 cells, pseudo-phosphorylation of MFN2 at any of the same three sites had equal suppressive effects on mitochondrial fusion, measured as the decrease in aspect ratio compared to WT MFN2 (Figure 2A, right panel). Indeed, even concomitant pseudo-phosphorylation of all three sites (MFN2 EDE) was not significantly more effective at impairing mitochondrial fusion than any of the single site phosphomimic mutants (Figure 2A, right panel). Thus, PINK1 phosphorylation of MFN2 at any or all of these sites suppresses mitochondrial fusion. Finally, mimicking phosphorylation at MFN2 S442 (--E) was a more potent inducer of Parkin translocation than doing so at T111 (E--) (Figure 2C, right panel; Figure 2D). Indeed, MFN2 --E and E-E were equally effective at promoting mitochondrial Parkin recruitment. Remarkably, mimicking phosphorylation at S378 had absolutely no effect on mitochondrial Parkin recruitment, either when T111 and S442 were also mutated to mimic phosphorylation or in isolation (-D-). Thus, phosphorylation of MFN2 T111 and S442, but not of S378, regulates mitochondrial Parkin recruitment.

### Dominant Functional Effects of MFN2 Phosphorylation Site Mutants Affect Mfn1 and Mfn2 Equally

**FIGURE 3:**
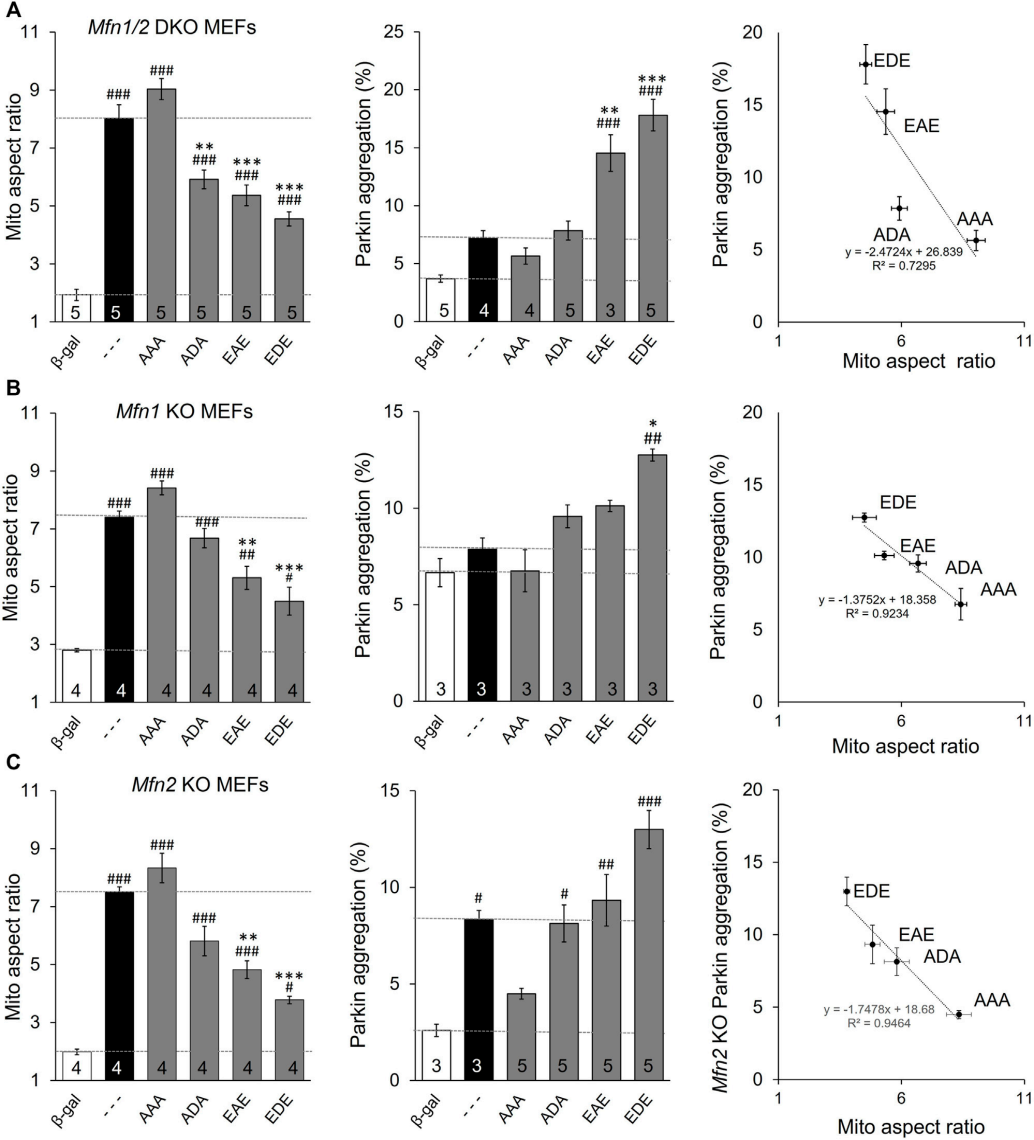
MFN2 PINK1 phosphorylation site mutants dominantly affect mitochondrial fusion and mitophagy in *Mfn1*, *MFn2*, or *Mfn1/Mfn2* null MEFs. MFN2 triple mutants designated as in Figure 1A were expressed by adenoviral transduction of *Mfn1*, *Mfn2*, or *Mfn1/Mfn2* null murine fibroblasts. Adeno β-gal was negative control; --- indicates no mutation at T111, S378 or S442 (i.e. WT MFN2). **(A)** Mitochondrial aspect ratio (left panel), Parkin recruitment (middle panel) and 2-dimensional plot of mitochondrial aspect ratio vs. Parkin aggregation as a function of MFN2 phosphorylation site (right panel) in *Mfn1/Mfn2* null MEFs. **(B)** Mitochondrial aspect ratio (left panel), Parkin recruitment (middle panel) and a 2-dimensional plot of mitochondrial aspect ratio vs. Parkin aggregation as a function of MFN2 phosphorylation site (right panel) in *Mfn1* null MEFs. **(C)** Mitochondrial aspect ratio (left panel), Parkin recruitment (middle panel) and a 2-dimensional plot of mitochondrial aspect ratio vs. Parkin aggregation as a function of MFN2 phosphorylation site (right panel) in *Mfn2* null MEFs. N shown is number of independent experiments. Mutant vs. adeno WT Mfn2: *p* < 0.05 *; *p* < 0.01 **; *p* < 0.001***; MFN2 vs. adeno β-gal: *p* < 0.05 #; *p* < 0.01 ##; *p* < 0.001 ###, all by ANOVA. Error bars are SEM.

The above data distinguish between MFN2 T111 and S442, whose phosphorylation status reciprocally regulates MFN2-mediated mitochondrial fusion and Parkin recruitment, and MFN2 S378 whose phosphorylation status regulates fusion, but not Parkin binding. However, all of the engineered mutants except triple site mutants MFN2 EDE and AAA retained native S or T that could be phosphorylated/de-phosphorylated by endogenous mechanisms, possibly activated as collateral effects of modulated fusion, depolarization, or Parkin recruitment. The ambiguous phosphorylation status of native amino acids in the single and double site mutants could be a confounding influence if, for example, there was cooperativity in phosphorylation between sites. For this reason, we created MFN2 mutants in which the phosphorylation status was concomitantly defined at all three sites (AAA, ADA, and EAE & EDE). The results of mitochondrial lengthening and Parkin recruitment assays for these mutants after their expression in mitofusin null cells are shown in Figure 3A. Although phosphorylation at S378 (ADA) has no significant effect on Parkin aggregation, the overall relationship between number of phosphorylation sites and fusion is inverse (Figure 3A, left panel) and with Parkin binding is direct (Figure 3A, middle panel). The reciprocal and mutually exclusive relationship between MFN2 functioning as a fusion protein vs. a Parkin binding protein is best illustrated by merging the results, as in Figure 3A, right graph.

MFN2 mutants expressed in *Mfn* double null cells reveal autonomous functioning of those mutants independent of endogenous Mfn1 or Mfn2. To determine how MFN2 phosphorylation site mutants impacted endogenous Mfn1 or Mfn2 functionality, the mutants were expressed in cells lacking either Mfn1 (i.e., expressing only Mfn2) (Figure 3B) or Mfn2 (and therefore expressing only Mfn1) (Figure 3C). The two *Mfn* null cell lines exhibited differences in basal mitochondrial aspect ratio and, especially, Parkin aggregation (Figures 3B,C middle panels, white bars). The latter is explained by absence of an Mfn2 Parkin receptor in fusion-defective *Mfn2* null cells, whereas fusion-defective *Mfn1* null cells can still mount an aggressive mitophagic response via Mfn2 Parkin receptors. Importantly, the relative effects of expressed MFN2 phosphorylation site mutants were the same in *Mfn1*-, *Mfn2*-, and *Mfn1/Mfn2*-double null cells. Two-dimensional plots of mitochondrial aspect ratio vs. Parkin aggregation as a function of MFN2 phosphorylation site status illustrates these relationships (Figures 3B,C, right graphs).

### Mitochondrial Parkin Recruitment Reflects Mitophagy

**FIGURE 4:**
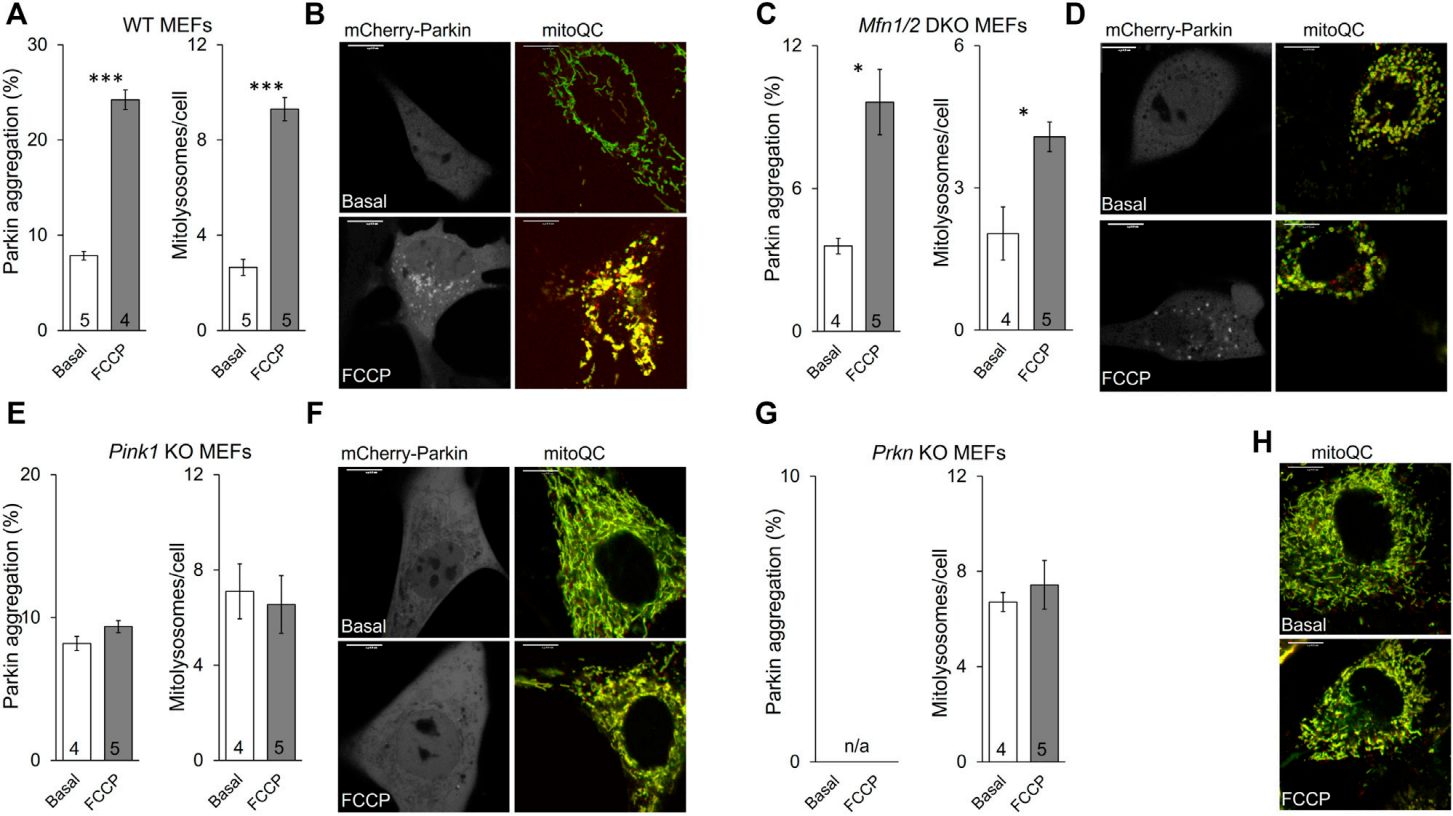
Mitochondrial Parkin recruitment and mitolysosome formation in cells lacking different mitophagy factors. **(A,B)**, wild-type (WT) MEFs; **(C,D)**, *Mfn1/2* DKO MEFs; **(E,F)**, *Pink1* KO MEFs; **(G,H)**, *Prkn* KO MEFs. Parkin aggregation was assayed by visualization of virally expressed mcParkin in cells untreated (basal) or treated with FCCP (5 μM, 1 h). Mitolysosome formation was measured with virally expressed mitoQC in cells untreated (basal) or treated with FCCP (5 μM, 8 h). **(A,C,E,G)** show mean group data. Basal vs. FCCP treatment: *p* < 0.05 *; *p* < 0.01 **; *p* < 0.001***, all by student’s t-test. **(B,D,F,H)** are representative images for each cell type; different cells for basal and FCCP-treated conditions. Supplementary Figure S2 has channel-separated images for mitoQC. Note that Parkin aggregation cannot be measured in *Prkn* KO MEFs (n/a). MFN2 mutant phosphorylation effects are recapitulated in PINK1 deficient cells.

The established role for Mfn2 in mitophagy is as a mitochondrial receptor for Parkin, recruiting it from cytosol after Mfn2 phosphorylation by PINK1 (Chen and Dorn, 2013; Gong et al., 2015). In this paradigm Mfn2 is a key signaling intermediate between PINK1 and Parkin. However, PINK1 can also directly phosphorylate Parkin and its ubiquitin substrate (Kane et al., 2014). To better resolve the specific role of PINK1-mediated Mfn2 phosphorylation in this convoluted pathway we planned to express the triple phospho-site Mfn2 mutants in cells lacking either upstream PINK1 or downstream Parkin. The experimental foundation for such studies was first laid by comparing mitochondrial Parkin recruitment, which is the initial step in Parkin-mediated mitophagy, to mitochondrial incorporation into lysosomes, which is the final step. The responses to mitochondrial depolarization with FCCP were compared between wild-type (WT) MEFs (Figure 4A) having all three key factors and cells lacking mitofusins (*Mfn1/Mfn2* DKO MEFs) (Figure 4B), MEFs lacking Pink1 (Figure 4C), and cells lacking Parkin (Figure 4D). As expected, the mitophagic response to FCCP was robust in WT MEFs, and mitochondrial Parkin recruitment was paralleled by mitochondrial incorporation into lysosomes (Figure 4A). Both Parkin aggregation and mitolysosome formation were greatly attenuated in mitofusin-null MEFs (Figure 4B, compare Y-axis values to WT), reflecting absence of the primary Parkin receptor (Mfn2) with partial compensation by other Parkin recruitment mechanisms. Neither Parkin recruitment nor mitolysosome formation were stimulated by FCCP in cells lacking Pink1 kinase, consistent with Pink1 stabilization mediating Parkin recruitment (Figure 4C). Finally, it was not possible to measure Parkin recruitment in cells lacking Parkin, but mitolysosome formation was not increased in FCCP-treated Parkin null MEFs (Figure 4D).

**FIGURE 5:**
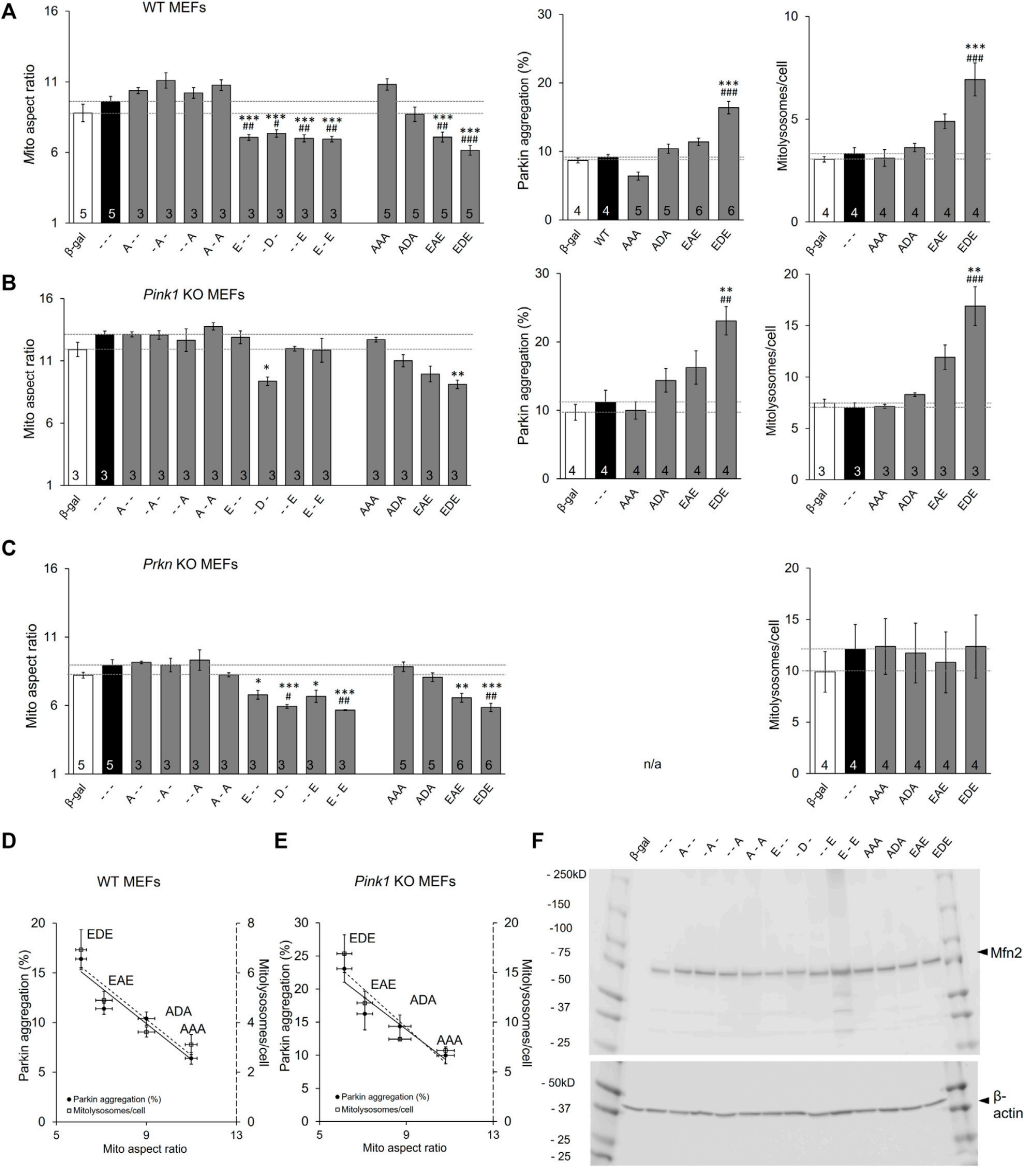
The roles of Pink1 and Parkin in Mfn2-mediated mitochondrial fusion and mitophagy. **(A)** Results of mitochondrial aspect ratio (left panel), MitoQC studies measuring mitolysosome number (middle panel) and Parkin aggregation (right panel) in WT MEFs. **(B,C)** Parallel studies in *Pink1* KO MEFs **(B)** and *Prkn* KO MEFs **(C,D,E)** AR vs. Mitophagy graph as a function of phosphorylation in WT MEFs **(D)** and *Pink1* KO MEFs **(E)**. **(F)**. Immunoblot analysis of adeno-MFN2 mutant expression in Mfn1/Mfn2 DKO MEFs. N shown is number of independent experiments. Mutant vs. adeno WT Mfn2: *p* < 0.05 *; *p* < 0.01 **; *p* < 0.001***; MFN2 vs. adeno β-gal: *p* < 0.05 #; *p* < 0.01 ##; *p* < 0.001 ###, all by ANOVA. Error bars are SEM.

*In vitro* phosphorylation with mass spectroscopy identified MFN2 phosphorylation by PINK1 kinase on T111, S378, and S442 (Rocha et al., 2018). To control for possible PINK1 kinase effects on other MFN2 sites or on other proteins we expressed MFN2 phosphorylation site mutants in MEFs derived from *Pink1* KO mice (Kitada et al., 2007). Basal mitochondrial aspect ratio was higher in *Pink*1 KO MEFs than WT MEFs (compare Figure 5A,B left panels), likely because abrogation of PINK1-mediated MFN2 phosphorylation interrupts the normal process for negative regulation of mitochondrial fusion in these cells (*vide supra*). In this context, only pseudo-phosphorylation at MFN2 S378 (-D-) suppressed mitochondrial fusion, measured as reduced aspect ratio (Figure 5B left panel), further supporting the idea that S378 is central to fusion modulation. However, the relationships between MFN2 phosphorylation site number, fusion, and Parkin recruitment were unchanged in absence of PINK1 kinase (Figure 5B). MitoQC-measured mitolysosome formation paralleled Parkin recruitment (Figure 5A,B middle and right panels).

### Parkin Is Dispensable for Regulation of MFN2-Mediated Mitochondrial Fusion

Taken together, the above results show that PINK1-mediated phosphorylation can be a negative regulator of MFN2-mediated mitochondrial fusion and, concomitantly, a positive regulator of PINK1-Parkin mitophagy. Although different phosphorylation sites have quantitatively different effects on this fusion/mitophagy switch, the general rule is strongly supported by the above data.

The phosphorylation mechanism suggested by our results contrasts with the commonly accepted idea that mitofusins are principally regulated by Parkin-mediated ubiquitination that stimulates selective proteasomal degradation (Neutzner and Youle, 2005; Cohen et al., 2008; Gegg et al., 2010; Glauser et al., 2011). Because PINK1 activity is a prerequisite for mitochondrial Parkin translocation and its ubiquitination of mitochondrial proteins (Kane et al., 2014; Koyano et al., 2014), it remained possible that PINK1 ablation interrupted Parkin-mediated MFN2 regulation by ubiquitination. We reasoned that expressing MFN2 phosphorylation site mutants in *Prkn* null cells could resolve this issue.

The familiar inverse relationship between MFN2 phosphorylation and mitochondrial elongation was unchanged in MEFs derived from mice genetically devoid of Parkin (Goldberg et al., 2003) (Figure 5A–C left panels). This was most evident for the triple phosphorylation site mutants, AAA, ADA, EAE, and EDE, in which simultaneous mutation of all three sites eliminated the possibility of their phosphorylation/de-phosphorylation by endogenous kinases and phosphatases. By contrast, the relationship between MFN2 phosphorylation and mitophagy, measured either as Parkin aggregation or downstream mitolysosome formation, was lost in the absence of Parkin (compare Figure 5A,C). Figures 5D,E summarize the relationship between MFN2 phosphorylation status in the triple phosphorylation site mutants on fusogenicity and mitophagy in WT and *Pink1* KO MEFs, respectively.

The above results demonstrate that Parkin is necessary for positive regulation of MFN2-facilitated mitophagy, but is dispensable for negative regulation of MFN2-mediated mitochondrial fusion. The putative Parkin-mediated ubiquitin/proteasome mechanism of fusion suppression dictates that negative regulation of mitochondrial fusion accrues from downregulation (by accelerated degradation) of MFN proteins (Neutzner and Youle 2005; Cohen et al., 2008; Gegg et al., 2010; Glauser et al., 2011). The possibility that our engineered MFN2 mutants were expressed at different steady-state levels, possibly from differential degradation, was rejected by comparative immunoblotting (Figure 5F).

## Discussion

These experiments identify site-specific functional consequences of Mfn2 phosphorylation by PINK1 kinase, and demonstrate that MFN2-mediated mitochondrial fusion is under binary “all or none” control. Mitochondrial fusion is fully enabled when Mfn2 is not phosphorylated, and is maximally suppressed when any of 3 PINK1 sites are phosphorylated. By contrast, MFN2-mediated recruitment of Parkin to mitochondria exhibited a phosphorylation site “dose” response, with each phosphorylation site contributing to a different extent. Thus, WT MFN2 and MFN2 AAA that is incapable of being phosphorylated evoked only low levels of Parkin recruitment. Pseudo-phosphorylation at T111 increased this, and pseudo-phosphorylation at S442 increased it even further. Importantly, MFN2 S378 seems uninvolved in MFN2-mediated mitochondrial Parkin binding.

To maintain respiratory fitness and produce ATP that fuels cellular functioning, mitochondria self-identify for repair, transportation, and replication or elimination. Outer mitochondrial membrane MFN proteins are central regulators of these processes, comprising a communications interface between resident mitochondria and host cells. Orchestration by MFN2 of fusion between healthy mitochondria, or of mitophagy that selectively removes damaged organelles, is essential to maintaining homeostatic balance (Shirihai et al., 2015; Gustafsson and Dorn, 2019). Thus, contextually appropriate regulation of MFN2 functioning is central to cell fitness. The current findings, using recombinant expression of MFN2 mutants engineered to mimic phosphorylated or non-phosphorylated MFN2 T111, S378, and S442 to interrogate MFN2 functioning in mitochondrial fusion and mitophagy, reveal that MFN2 phosphorylation at these sites is sufficient to direct its activity to fusion or mitophagy.

These data suggest a need to re-evaluate previous conclusions that mitofusins are regulated primarily through Parkin-mediated ubiquitination and selective proteasomal degradation. Given the recognized importance of mitofusin regulation to cell health (Detmer and Chan 2007), it seems surprising that pathophysiologically relevant mechanism(s) regulating MFNs have not been unambiguously defined. Conventional wisdom is that Parkin-ubiquitylated mitofusins undergo proteasomal degradation (Tanaka et al., 2010; Anton et al., 2013; Escobar-Henriques and Joaquim, 2019). But, Parkin ubiquitinates many mitochondrial proteins (Sarraf et al., 2013), and it is not clear how the proteasome system could specifically remove ubiquitinated mitofusins from the outer membranes of mitochondria separately from a hundred or more poly-ubiquitinated proteins. Nor is it clear how ubiquitination can fine-tune relative MFN activity in fusion and mitophagy. Our findings support the counter-hypothesis that PINK1 kinase directly phosphorylates MFNs, thereby modulating activity for fusion vs. mitophagy.

While our studies should help settle the controversy about whether Mfn2 functionality is predominantly regulated via post-translational modification vs. physical removal and degradation, our results raise a circumstantial question that requires additional investigation. In the context of mitochondrial quality control, the Mfn phosphorylation switch makes intuitive sense: Healthy mitochondria destabilize Pink1, maintaining Mitofusins in a non-phosphorylated state wherein they can fuse with their neighbors. Damaged mitochondria lose the ability to degrade Pink1, which accumulates and phosphorylates Mfn2 and other substrates, sequestering damaged organelle by turning off fusion, and recruiting Parkin that initiates targeted mitophagic removal. However, the Mfn2-Parkin signaling nexus has a developmental function in embryonic hearts wherein the transition from a predominantly carbohydrate-based metabolism to one wherein fatty acids are primarily metabolized is accomplished via Mfn2-Parkin mediated mitophagy (Gong et al., 2015). In this context, mitophagy-incompetent Mfn2 A-A prevented the normal mitophagic replacement of embryonic with adult mitochondria, resulting in a perinatal metabolic mismatch in myocardial muscle that proved incompatible with life. This does not seem likely to be a Pink1 kinase-mediated response. But, if not Pink1 then what is the developmentally-regulated kinase that performs the regulatory function in context of metabolic maturation of perinatal myocardium? Another question raised by these studies is whether there are mitochondrial phosphatases that oppose Pink1 (and perhaps other kinase) activity on Mfn2, to reverse the fusion/mitophagy switch.

In summary, as a general rule we observed that non-phosphorylated MFN2 was fusogenic, whereas mimicking phosphorylation on T111, S378 or S442 promoted mitochondrial Parkin recruitment and initiated mitophagy. However, the phosphorylation status at different sites variably impacted MFN2 functioning. Thus, our results point to a major role for phosphorylation of MFN2 S378 in disabling mitochondrial fusion, while this residue had little impact on Parkin recruitment. By contrast, mimicking phosphorylation of T111 or S442 was as effective as S378 in suppressing mitochondrial fusion, but S442 in particular enabled mitochondrial Parkin translocation. These differences within a general theme are consistent with the idea that MFN2 functioning is determined in large part by its binding affinity for different protein partners (Dorn, 2020): Mitochondrial fusion depends upon MFN-MFN partnering as cis- and trans-oligomers (Koshiba et al., 2004; Dorn, 2019), whereas mitophagy is initiated by MFN-Parkin binding (Chen and Dorn 2013). Although different MFN2 protein structure and interactive domains have been proposed (Koshiba et al., 2004; Franco et al., 2016; Mattie et al., 2017; Li et al., 2019; Franco et al., 2020), evidence is accumulating to support cis interactions that form toroidal MFN oligomeric structures on different mitochondria and interact in trans to tether and fuse organelle outer membranes (Liu et al., 2018; Dorn 2020). Certainly, there is abundant precedent for phosphorylation as a mechanism to direct protein-protein binding. The most famous example of this paradigm is as a determinant of cell signaling for superfamily of 7-transmembrane spanning GTP-coupled membrane receptors. Agonist-occupied unphosphorylated receptors couple to heterotrimeric GTPases, but dissociate from these GTPases after phosphorylation by G-protein receptor kinases (GRKs) and instead couple to β-arrestins (Lefkowitz, 2007; Ribas et al., 2007; Mayer et al., 2019). Our data demonstrate how mitofusins also follow this paradigm.

## Data Availability

The original contributions presented in the study are included in the article/Supplementary Material, further inquiries can be directed to the corresponding author.

## Supplementary Material

Supplementary Figure S1 Parkin is recruited to mitochondria upon FCCP treatment of WT MEFs. Left panel: at baseline, mcParkin (Mayer et al) appears diffusely cytosolic, and the mitochondrial have normal morphology (green). Right panel: after FCCP treatment (5 µM, 1 hour), Parkin translocates to mitochondria, forming visible aggregates. Also note mitochondrial fragmentation after FCCP.

Supplementary Figure S2 Mitochondrial mitolysosome formation in cells lacking different mitophagy factors. This figure is an expanded version of representative mitoQC images from Figure 4. **(A,B)** wild-type (WT). **(C,D)** *Mfn1/2* DKO, **(E,F)** *Pink1* KO or **(G,H)** *Prkn* KO MEFs. **(A,C,E,G)**, basal condition. **(B,D,F,H)**, FCCP treated condition. Left panels are red images from mitoQC mCherry signal, middle panels are green images from mitoQC EGFP signal, right panels are merged mitoQC image.
