# Supplementary figures and images for: Reciprocal Regulation of Mitofusin 2-Mediated Mitophagy and Mitochondrial Fusion by Different PINK1 Phosphorylation Events
Source: Front Cell Dev Biol. 2022 May 12;10:868465. doi: 10.3389/fcell.2022.868465 (PMC9133611; doi:10.3389/fcell.2022.868465)

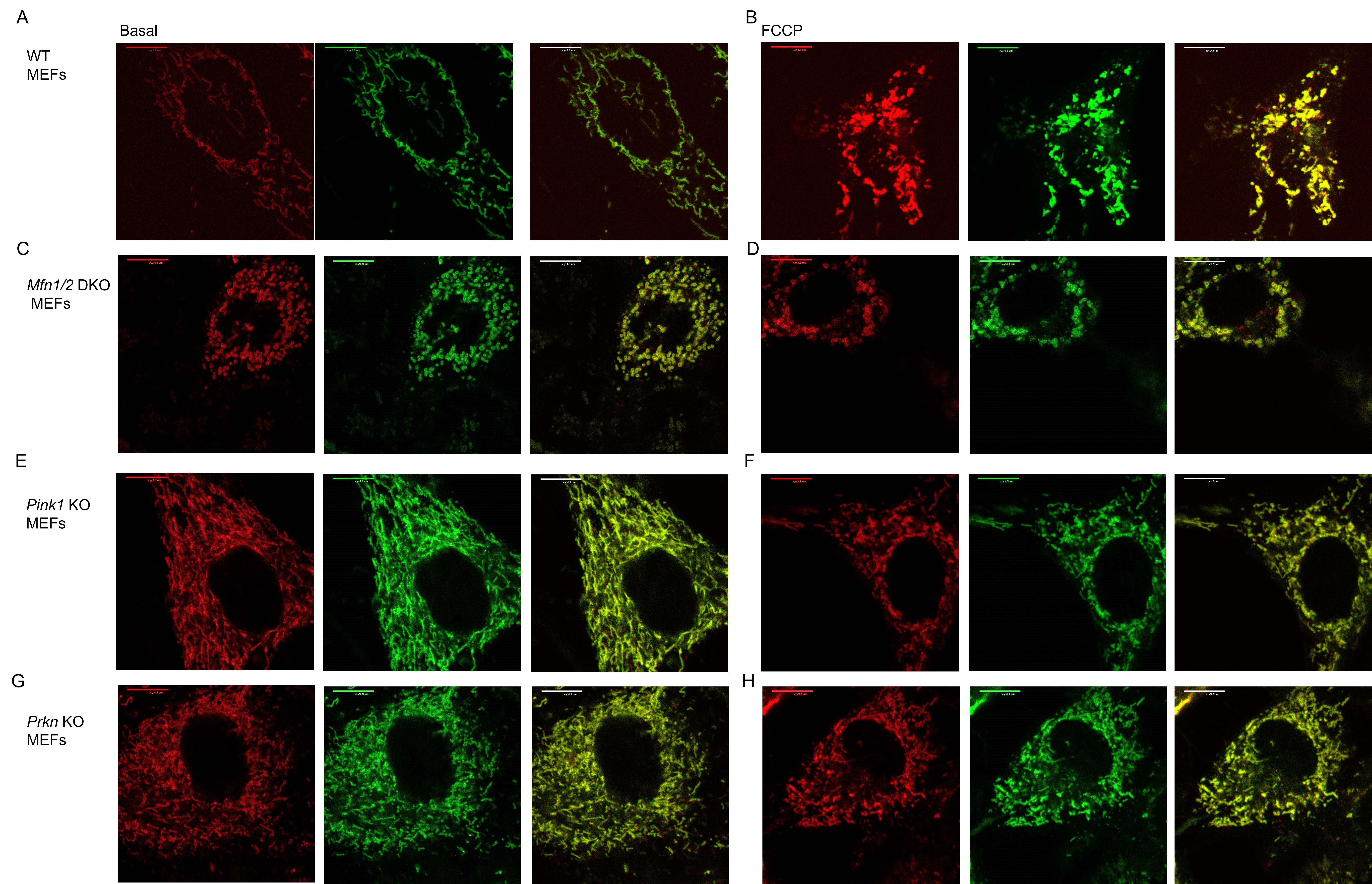

Supplement: Supplementary file 1 [file Image2.JPEG]

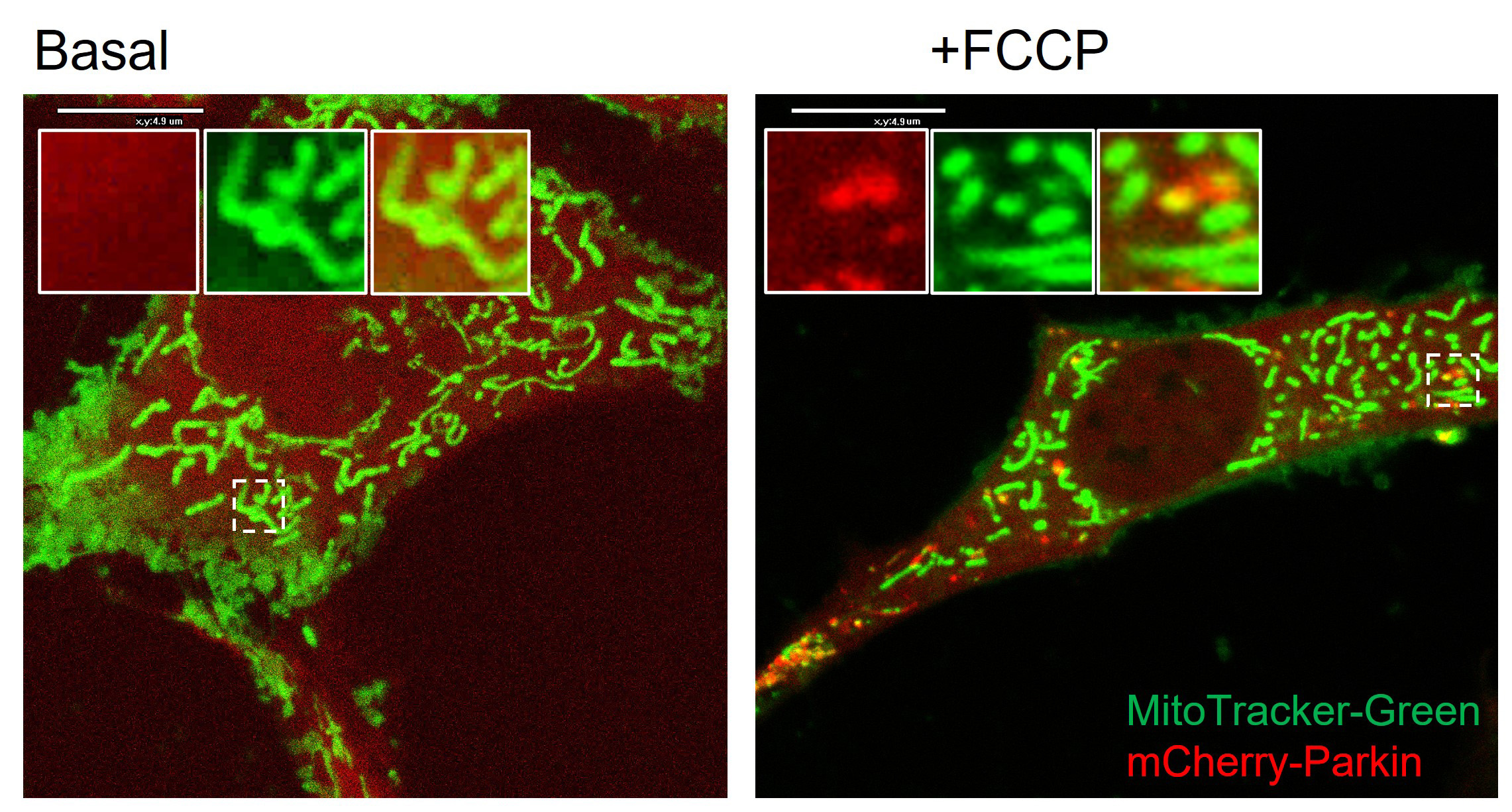

Supplement: Supplementary file 2 [file Image1.jpg]
